# Optical Limiting of Carbon Nanohorn-Based Aqueous Nanofluids: A Systematic Study

**DOI:** 10.3390/nano10112160

**Published:** 2020-10-29

**Authors:** Elisa Sani, Nicolò Papi, Luca Mercatelli, Simona Barison, Filippo Agresti, Stefano Rossi, Aldo Dell’Oro

**Affiliations:** 1 CNR-INO National Institute of Optics, Largo E. Fermi, 6, I-50125 Firenze, Italy; nicolo.papi@tutanota.com (N.P.); luca.mercatelli@ino.it (L.M.); 2 CNR-ICMATE Institute of Condensed Matter Chemistry and Technologies for Energy, Corso Stati Uniti, 4, I-35127 Padova, Italy; simona.barison@cnr.it (S.B.); filippo.agresti@cnr.it (F.A.); 3 CNR-ITC Institute of Construction Technologies, Corso Stati Uniti, 4, I-35127 Padova, Italy; stefano.rossi@itc.cnr.it; 4 INAF, Astrophysical Observatory of Arcetri, Largo E. Fermi, 5, I-50125 Firenze, Italy; aldo.delloro@inaf.it

**Keywords:** carbon nanohorns, nanofluids, optical limiting

## Abstract

Nowadays, the use of lasers has become commonplace in everyday life, and laser protection has become an important field of scientific investigation, as well as a security issue. In this context, optical limiters are receiving increasing attention. This work focuses on the identification of the significant parameters affecting optical limiting properties of aqueous suspensions of pristine single-wall carbon nanohorns. The study is carried out on the spectral range, spanning from ultraviolet to near-infrared (355, 532 and 1064 nm). Optical nonlinear properties are systematically investigated as a function of nanohorn morphology, concentration, dimensions of aggregates, sample preparation procedure, nanostructure oxidation and the presence and concentration of surfactants to identify the role of each parameter in the nonlinear optical behavior of colloids. The size and morphology of individual nanoparticles were identified to primarily determine optical limiting. A cluster size effect was also demonstrated, showing more effective optical limiting in larger aggregates. Most importantly, we describe an original approach to identify the dominant nonlinear mechanism. This method requires simple transmittance measurements and a fitting procedure. In our suspensions, nonlinearity was identified to be of electronic origin at a 532 nm wavelength, while at 355 nm, it was found in the generation of bubbles.

## 1. Introduction

Optical limiting systems are extremely important to protect human eyes or instrumentation in all applications where high-power laser beams are involved. Optical limiting (OL) is referred to as the phenomenon shown by some materials where the energy transmitted through the sample follows two well distinct regimes; at weak input intensities, the light transmitted through the material linearly depends on the intensity of incident radiation. In these conditions, the value of optical transmittance is constant and independent of the input light intensity. However, when the intensity of input light becomes higher than the threshold value characteristic of the material, the intensity of transmitted light becomes less dependent than linear on the input light, becoming also, in ideal cases, constant and independent of the incident intensity. In this regime, the transmittance suddenly decreases with respect to its initial low-intensity value, reaching, for high input intensities above the threshold, values even near to zero. In these conditions, the material becomes opaque or near-opaque to the incident high-intensity radiation. An extremely wide range of OL materials has been described in the literature, including organic molecules like porphyrins and phthalocyanines [1]; bulk solids, e.g., semiconductors [2]; liquid crystals films [3]; and a variety of different nanoparticles both suspended in liquids (see, for instance, [4] for gold and silver nanoparticles of different shapes) and inserted in solid matrices [5]. Among nanoparticle-based systems, the family of carbon allotropes has received great interest, starting with early works on fullerenes in the 1990s [6,7]. From then, various carbon-based nanoparticles, such as nanotubes [8], nano-onions and nanodiamonds [9], have been proved to show OL. In the majority of cases, nanoparticles were suspended in fluids, thus actually producing a system that later was named as a nanofluid [10], i.e., a fluid where nanometer-sized particles are dispersed with the aim of changing the functional properties of the base fluid. The interest in nanofluids has hugely grown over the last years due to the rapid development of nanotechnology arising in the availability of new nanoparticles and in the development of techniques to disperse them in fluids with improved stability. Nanofluids are of interest in many application fields, as they exhibit solid-like properties in a liquid phase. Major applications include heat transfer and energy [11], among others. The study of nanofluids also gave an important contribution to the development of advanced modelling techniques, e.g., artificial neural networks [12]. Carbon-based nanofluids, in particular those containing nanoparticles belonging to the family of carbon nanotubes (CNTs), have been widely studied for their electronic and thermal properties [13]. Moreover, they also show some good optical limiting characteristics, as mentioned above. Unfortunately they also have a critical drawback due to their toxicity [14], which largely reduces possible applications and motivates the research towards alternative nanoparticles. 

Single-walled carbon nanohorns (SWCNHs) were discovered by S. Ijima in 1999 [15]. They show a horn-shaped wrapping of a single graphene sheet with a 30–50 nm length and a 2–5 nm diameter. Differently from the CNTs family, SWCNHs have non-toxic or non-carcinogenic effects in animals [16]. For this reason, new possible applications have been proposed for them, e.g., biosensors, drug delivers, gas storage [17] or, in the solar energy sector, direct sunlight absorbers [18]. Recently, OL at wavelengths of 1064 and 532 nm was demonstrated for SWCNH dispersed in acetone [19], without, however, the provision of many details about the investigated samples. For this reason, the characterization of the basic properties of these intriguing carbon allotropes being our goal, we focused our study on the systematic investigation of several nanoparticle and colloid parameters (nanohorn morphology, concentration, dimensions of aggregates, sample preparation procedure, and the presence and concentration of surfactants). In addition, we prove the nonlinear behavior of carbon nanohorns under irradiation by ultraviolet light at 355 nm, i.e., exciting the nanostructures near their plasmonic peak, obtaining an effect more pronounced than that previously obtained at 532 and 1064 nm wavelengths. Moreover, the novelty of the present report is severalfold: (1) to assess, for the first time, whether and how different colloid parameters affect the obtained nonlinear properties to provide deeper insight into the fundamental properties of the system; (2) to demonstrate OL in water-based suspensions, with the aim to promote the use of environmentally friendly materials and to widen their application spectrum. In fact, it is known that OL in nanofluids strongly depends on the base fluid, as observed on graphene [20] and single-walled nanotube suspensions [21,22]. Furthermore, a significant novelty of the work is (3) showing an original approach for optical limiting studies based on a simple experimental setup combined with literature models, which allows us to assess the dominant mechanism producing the nonlinear behavior. The mechanisms disclosed with our method agree with those arising from direct measurements [19], confirming the validity and reliability of our simple approach. Finally, (4) the formation of vapor bubbles due to interaction with laser photons that we documented widens the future applications of SWCNH-based aqueous nanofluids to direct solar vapor generation and solar desalination systems. 

## 2. Experimental 

Several suspensions of SWCNHs in water were prepared to study whether and how the optical properties of interest are connected to different sample parameters, such as nanofluid preparation procedure, nanoparticle concentration, nanoparticle morphology and dimensions of aggregates in the fluid. The prepared samples are identified by labels as follows:

(a) The series of NH1–NH5 samples was made by preparing a solution containing 0.005 g/L of sodium dodecyl sulphate (SDS) (99%, provided by Sigma-Aldrich (St. Louis, MI, USA) in water, and 0.05 g/L pristine SWCNHs with dahlia-like morphology and a 60 nm mean size (provided by Carbonium S.r.l., Selvazzano Dentro, Italy) were pre-dispersed in the solution by sonication with a VCX 130 ( Sonics & Materials, Newtown, CT, USA) ultrasonic processor operating at 20 kHz and 65 W for 10 min. The final optimized dispersion was obtained by high-pressure homogenization using a Panda (GEA Niro Soavi, Parma, Italy) operated at 1000 bar for different times. Sample NH1 corresponds to the only sonicated suspension, while samples NH2, NH3, NH4, NH5, were also homogenized for 15, 30, 45, 60 min respectively. The obtained sample was further diluted with distilled water to obtain lower concentrations as indicated in Table 1. 

(b) To prepare the functionalized sample by oxidation, labelled as OX, 0.05 g of the same SWCNH powders as in (a) was poured into 25 mL HNO3 (68–70%, provided by Alfa Aesar (Heysham, UK) and stirred for 2 h at 80 °C to obtain proper surface oxidation. The suspended powders were separated from HNO_3_ by filter paper and thoroughly washed with deionized water until the filtrate reached neutral pH. Finally, the powders were washed with 100 mL ethanol (absolute anhydrous, provided by Carlo Erba, Cornaredo, Italy) and dried at 120 °C for 2 h. Colloidal suspensions of the treated nanopowders at the concentration of 0.05 g/L were prepared by pouring 0.0125 g of oxidized powder in 250 mL deionized water, and the suspensions were sonicated for 10 min at 20 kHz and 65 W. The final colloidal suspensions were obtained by processing the sonicated suspensions with a high-pressure homogenizer at 1000 bar for 15 min. 

(c) The B80nm and D80nm samples, characterized by a different size (80 nm) than the SWCNH powders previously described in (a) and (b), and differing from each other in morphology (B80nm: bud-like and D80nm: dahlia-like), were prepared mechanically dispersing pristine SWCNHs in a water solution of SDS. An ultrasonic processor (VCX 130, Sonics & Materials) (Newtown, CT, USA) operating at 20 kHz and 70 W for 30 min was employed to obtain stable suspensions. 

Particle size and zeta potential were measured using a Zetasizer Nano ZS (Malvern Panalytical, Malvern, UK) based on dynamic light scattering (DLS).

**Figure 1:**
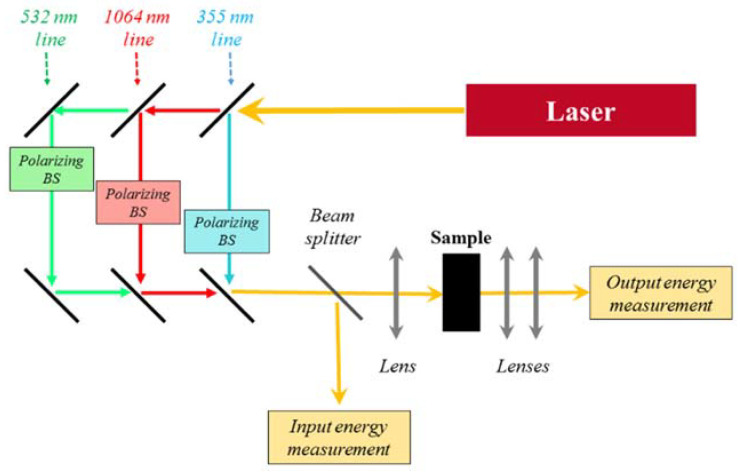
Experimental setup for optical limiting experiments.

Spectral optical transmittance in the linearity regime was measured for diagnostic purposes using a double-beam UV–VIS spectrophotometer (Lambda900, PerkinElmer, Waltham, MA, USA) using a variable length cell [23,24]. High intensity light irradiation experiments were carried out using a pulsed nanosecond Nd:YAG laser as light source (Q-smart 850, Quantel lasers, Les Ulis, France, delivering 6 ns pulses at 1064, 532 and 355 nm wavelengths with a 10 Hz repetition rate). The three laser emission wavelengths were spatially separated by proper optical elements and focused on the sample by a lens. The sample was held in a quartz cuvette with a 10 mm path length. The beam exiting the sample was collected by a couple of lenses and focused on a pyroelectric detector (PE25C, Ophir Optronics, Jerusalem, Israel). The energy incident on the cuvette could be varied by means of proper polarizer beam splitters and measured using a pyroelectric detector (PE50BF, Ophir Optronics, Jerusalem, Israel) (Figure 1). As a common protocol, all the samples were sonicated in an ultrasonic bath (ProsKit SS803F) (Prokit’s Industries Co., Ltd., New Taipei City, Taiwan) for 10 min before the measurements.

## 3. Results and Discussion

All the investigated samples are listed in Table 1. Several subgroups can be identified among them, and they can be discriminated by different parameters: preparation technique (groups NH-OX-D80nm), aggregate morphology (dahlias with an 80 nm dry size: sample D80nm; dahlias with a 60 nm dry size: NH series samples and OX; buds with an 80 nm dry size: sample B80nm), dimensions of aggregates (NH series, see Table 2 below), etc. Moreover, each sample was prepared in different concentrations depending on the overall linear absorption at the investigated wavelengths. 

Mean dimensions of aggregates in fluid (DLS hydrodynamic sizes, measured by the relationship between the size of particles and their speed due to Brownian motion through the Stokes–Einstein equation), and ζ potential were measured to evaluate the aggregation and the stability of the suspensions. The absolute value of the ζ potential indicates the degree of electrostatic repulsion between adjacent, similarly charged particles in dispersion. A colloid is usually considered stable if the absolute value of zeta-potential is higher than 30 mV. The results are summarized in Table 2. In the NH1–NH5, series it is clear that the homogenization process decreases the aggregate dimension and improves the colloid stability up to an optimized homogenization time, as all homogenized samples have ζ-potential close to or higher than 30 mV in the absolute value. In fact, these samples in static conditions were stable for months; the pristine NH6 and the functionalized OX samples even showed higher ζ-potential values, indicating a very high stability.

**Figure 2:**
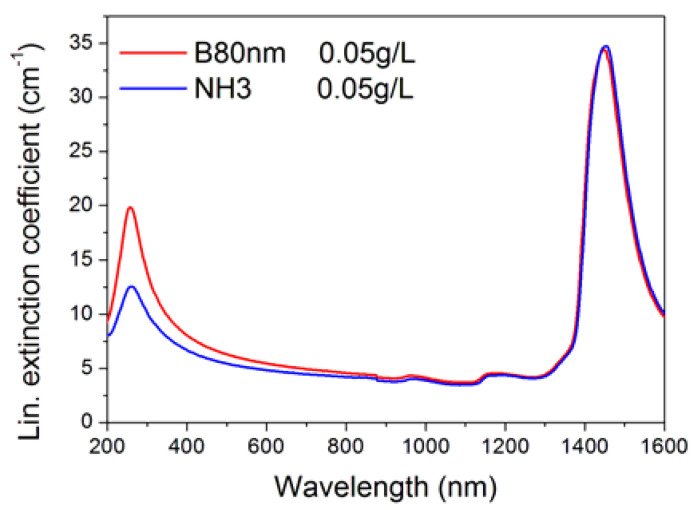
Spectral linear extinction coefficient of samples B80nm and NH3 for the same 0.05 g/L concentration.

From the transmittance spectra, we obtained the extinction coefficient of samples in the linear regime, i.e., at low input light intensity. Figure 2 shows an example of the typical shape of the spectral curve for the B80nm and NH3 samples at 0.05 g/L concentration. The UV plasmonic peak at around 260 nm is characteristic of SWCNHs and extends towards infrared with an extremely smoothly decreasing tail. Spectral features at around 1000 and 1200 nm and, especially, the high peak at around 1445 nm can be ascribed to water. It is worth to mention that, even if the nominal concentrations of nanoparticles in the majority of investigated samples were the same, the specimens showed differences in the actual value of the extinction coefficient. This is connected to the intrinsic stability of each sample, which critically depends on the preparation technique, and on the connected occurrence of clustering and settling phenomena. In fact, if the nanofluid experiences clustering/settling, a part of the value of the extinction coefficient, that arising from nanoparticles, decreases. 

### 3.1. Broadband Optical Limiting

**Figure 3:**
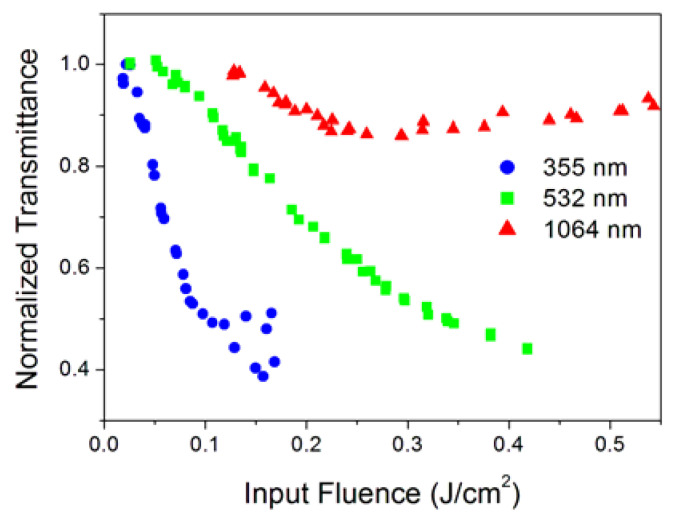
Normalized transmittance of the NH4 sample at 0.025 g/L as a function of the input energy spatial density (input fluence).

Almost all investigated nanofluids showed a typical optical limiting at all three laser wavelengths (confirming the desirable broadband behavior) similar to what is reported in Figure 3, where we can see an example for sample NH_4_. Figure 3 shows the normalized transmittance, defined as the ratio between the output and input energies E_out_/E_in_, normalized to its value at low incident intensities, e.g., in the linearity regime, as a function of the input fluence. 

In Figure 3 we can clearly distinguish three different regimes: at very low incident fluence, the behavior of the sample is linear (E_out_/E_in_ ≈ 1); after a certain input fluence value, the sample shows a decrease in the normalized transmittance—this is the optical limiting regime; and at higher incident energy densities, damage to the nanofluid can occur, and the normalized transmittance rises up again, as shown in Figure 3, for the 1064 nm wavelength, above 0.26 J/cm^2^. The damage to the nanofluids, when occurring, strongly depends on nanoparticle concentration and incident wavelength. Typically, the infrared radiation tends to damage the samples at lower energy compared to ultraviolet and visible wavelengths.

Nearly all the SWCNH samples we analyzed show broadband optical limiting qualitatively similar to that represented in Figure 3, except in the case of a few samples at the 1064 nm wavelength, where the quick damage at high input energies prevented acquiring enough experimental points to quantitatively characterize the nonlinear behavior. Table 3 shows the minimum obtained transmittances relative to the initial (low-energy) linear values. We can see that OL is more effective (i.e., a lower relative transmittance can be obtained) at shorter wavelengths due to the higher linear extinction coefficient.

### 3.2. Effect of Surfactant Concentration

**Figure 4:**
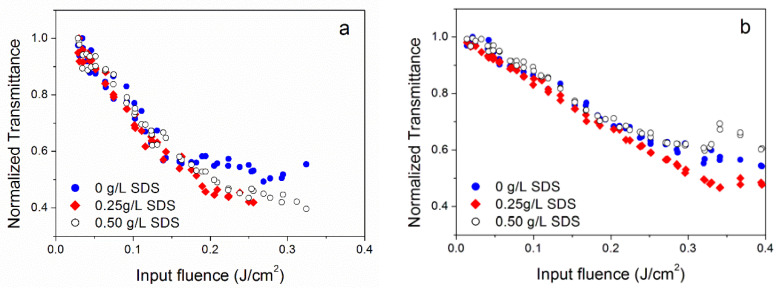
Effect of the surfactant concentration on the optical limiting properties of the NH6 sample at 355 (**a**) and at 532 nm (**b**).

For all nanofluids, with the only exception of OX, the SDS surfactant was added during the preparation (Table 1). Therefore, initially, we checked that the observed OL in nanofluids would not be directly ascribed to the surfactant itself. The experiments carried out on the base fluid with the surfactant, but with no nanoparticles, are reported in the Appendix A and did not show a nonlinear effect in the absence of nanoparticles. It could it be possible that the surfactant has a role in modifying the nonlinear properties of nanofluids as it changes the van der Waals interaction between nanoparticles and the surface tension of the base fluid. To check this, we investigated a series of samples with the same nanoparticle type and concentration and different SDS concentrations. Figure 4 shows the optical limiting properties of the NH6 nanofluid (0.0125 g/L SWCNH concentration) changing the concentration of the surfactant from 0 to 0.50 g/L under UV and visible radiation. 

For neither for the 355 nor 532 nm wavelength was it possible to identify an effect of SDS concentration on the optical limiting properties of SWCNH-nanofluids, and a similar result was found for incident radiation at 1064 nm. The effect of the surfactant was only detectable, as expected, when the damage threshold of the samples was concerned. For the 355 nm wavelength, the lowest stability sample was that without the surfactant, while at 532 and 1064 nm wavelengths, the most stable were those with intermediate 0.25 g/L SDS content. This result suggests that, for each wavelength, an optimal concentration of the surfactant exists that maximizes the nanofluid stability under high-intensity optical radiation at that wavelength, which is an interesting result in view of future applications. 

### 3.3. Effect of Nanoparticle Concentration

**Figure 5:**
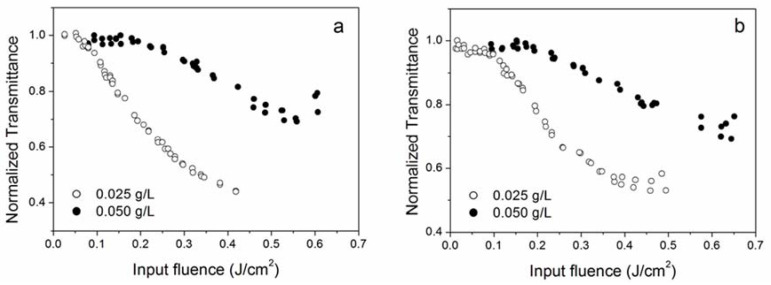
Optical limiting curves for NH4 (**a**), and NH5 (**b**) samples at 532 nm at different nanoparticle concentrations.

**Figure 6:**
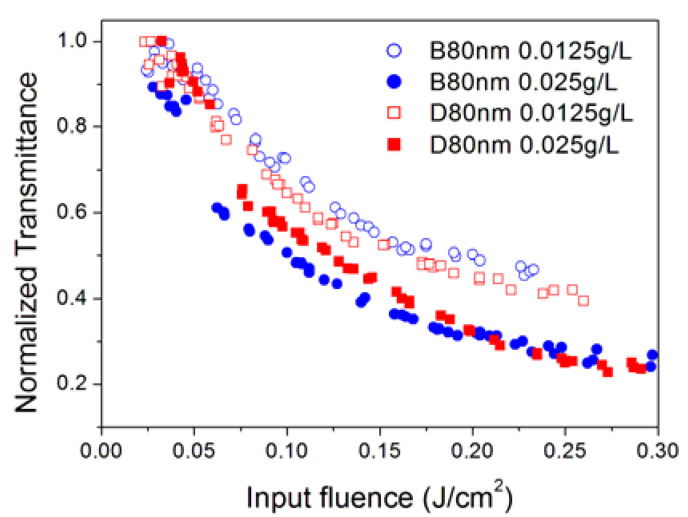
Optical limiting at 355 nm for B80nm and D80nm samples at two different nanoparticle concentrations.

The optical limiting properties of nanofluids also depend on the concentration of nanoparticles. All sample types, except NH6, were studied at two different nanoparticle concentrations. Due to the high linear extinction coefficient, for the characterization at 355 nm, it was needed in some cases (B80nm, D80nm, OX) to prepare an additional specimen with a reduced concentration (0.0125 g/L). At 532 nm incident wavelength, optical limiting is more effective (i.e., it occurs with a higher slope as a function of the input energy) at a low nanoparticle concentration, as reported in Figure 5 for the samples NH4 and NH5. The same result holds true for the 1064 nm wavelength. This behavior suggests that OL in these cases could be due to a saturation effect of the first excited state (excited state absorption—ESA, see below), because if a larger number of nanoparticles exists in the light-pumped volume, more energy is required to saturate their excited level; thus, the optical limiting at high concentrations needs a higher incident energy to reveal itself. Differently, at 355 nm, better optical limiting properties were observed for higher nanoparticle concentrations, as shown in Figure 6. This behavior agrees with that reported in the literature for carbon nanotubes [25], graphite/nanodiamond mixtures [26], functionalized graphene nanoplatelets [27,28] and gold nanoparticles [29] and can be explained in terms of the light-induced creation of bubbles within the fluid. In fact, under the effect of light irradiation, nanoparticles become localized heat sources within the fluid, ultimately producing vaporization of the surrounding fluid layers and/or ionization and expansion of nanostructures themselves. Therefore, as the number of nanoparticles in the light-pumped fluid volume increases, more bubbles will be created by the laser, resulting in a larger decrease in the sample transmittance. 

### 3.4. Effect of Nanoparticle Morphology

**Figure 7:**
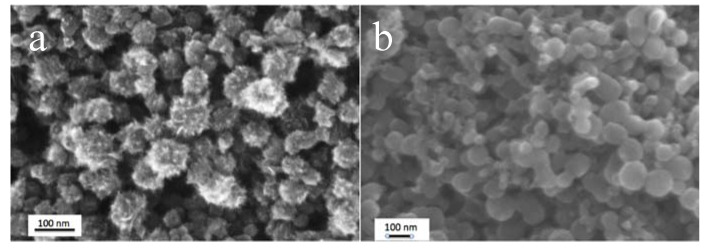
SEM images of dahlia (**a**) and bud (**b**) morphologies.

Depending on the synthesis conditions of nanopowders, it is possible to obtain some typical morphologies of their aggregates. In particular, if SWCNHs are produced by laser ablation, aggregates assume a typical appearance called “dahlia”. Otherwise if they are produced by arch discharge, their aggregation form tends to be a different shape called “bud-like” [30,31]. In the case of production by induction heating, as in the process used in our case, depending on the production parameters, either dahlia or bud morphologies ca be obtained. Figure 7 shows SEM images of our nanohorn powders. More details including TEM images, Raman and XRD investigations can be found in [32].

**Figure 8:**
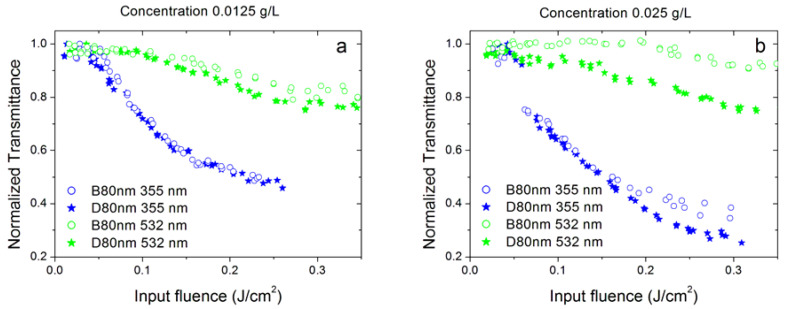
Optical limiting properties of the sample D80nm (dahlia—80 nm) and B80nm (bud—80nm) at 355 and 532 nm wavelengths, for nanoparticle concentration of 0.0125 (**a**) and 0.025 g/L (**b**).

**Figure 9:**
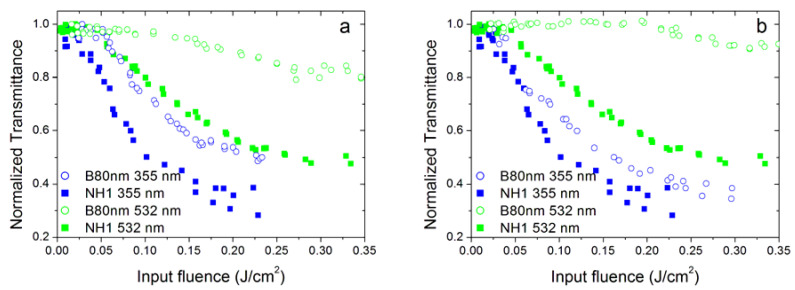
Optical limiting properties of the NH1 sample (dahlia) and B80nm (bud) at the 355 and 532 nm wavelengths: (**a**) for similar values of the linear extinction coefficient (corresponding to nanoparticle concentrations of 0.025 and 0.0125 g/L for dahlias and buds, respectively and (**b**) for the same nanoparticle concentration for both samples (0.025 g/L).

We investigated several samples belonging to the family of dahlias and a sample of bud morphology. Figure 8 and Figure 9 compare OL performances for samples belonging to dahlia and bud families. In particular, Figure 8 compares, at the same concentration and linear extinction coefficient values, dahlia and bud morphologies with similar mean dimensions of the starting dry nanoparticles (80 nm, labelled as samples D80nm and B80nm, respectively, in Table 1). It should be noted that these sizes are referred to as dry nanoparticles immediately after the synthesis and depend on the process parameters. When suspended in the fluid, individual nanoparticles can aggregate, producing clusters with the dimensions given in Table 2 (that include also the surfactant size), which, for these two samples, are 197 nm (buds) and 241 nm (dahlias), respectively.

From Figure 8, we can see that the properties of the two samples are quite similar, with dahlias slightly better performing than buds, especially at the 532 nm laser wavelength and at the highest concentration. However, the samples also showed different cluster dimensions, thus, this comparison is not sufficient to assess whether the observed differences could be a morphology or a cluster size effect.

For this reason, we also compared two dahlia/bud samples with similar cluster sizes (Figure 9): NH1 (dahlia with a cluster size of 202 nm) and B80nm (bud with a cluster size of 197 nm, see Table 2). The comparison was carried out considering two different situations. In the first case (Figure 9a), we considered different concentrations of the two samples in order to have similar values of linear extinction coefficients, and we found that the dahlia sample performed better at both 355 and 532 nm, and the same holds true for the 1064 nm wavelength. Numerical fitting (see the paragraph below) also confirmed the lowest energy threshold values shown by the sample (NH1, 0.025 g/L) with respect to B80nm (0.0125 g/L). In the second case (Figure 9b), we compared the two samples at the same concentration of 0.025 g/L. Again, the dahlia morphology showed stronger nonlinearity than buds, in particular for the green wavelength.

**Figure 10:**
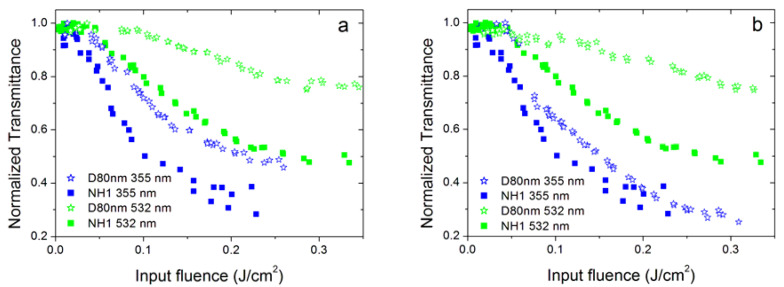
Optical limiting properties of the NH1sample (dahlia—60 nm) and D80nm (dahlia—80 nm) at 355 and 532 nm wavelengths: (**a**) for nanoparticle concentrations of 0.025 and 0.0125 g/L, respectively, and (**b**) for the same concentration of 0.025 g/L for both samples.

To complete the study of the dependence of optical limiting properties on nanoparticle morphology, a further comparison could be made between dahlia-like samples with different sizes of individual dry particles. Figure 10 compares the OL curves at 355 and 532 nm wavelengths of dahlias with individual average size of 80 nm (D80nm sample) and 60 nm (NH1 sample). Similarly to the previous case shown in Figure 9, a comparison was carried out either for similar values of extinction coefficients (Figure 10a) or with equal nanoparticle concentrations (Figure 10b). It is clear that OL is always more pronounced for dahlias with an average size of 60 nm (NH1), despite the fact that the cluster size is larger for the other D80nm sample (241 versus 202 nm, see Table 2). Interestingly, the largest differences in OL performances among samples are shown again at the 532 nm laser wavelength. As a final comment, we should note that the D80nm nanofluid shows better stability against damage, especially at 355 nm, in agreement with the measured values of the ζ-potential. 

The analysis carried out in the previous paragraphs (see Figure 8, Figure 9 and Figure 10) allows us to infer that the main parameters affecting OL properties are primarily the size of individual nanoparticles and, secondly, the nanoparticle morphology. The role of cluster dimensions seems of secondary importance when compared to the two previous parameters, but it can be identified when the other parameters are kept unchanged (see Section 3.5 below). 

It is worth noting that it is known that size and morphology are strictly connected to fundamental properties of nanoparticles, as they affect their energy level structure. Therefore, the largest impact of these parameters can be expected on the phenomena whose main origin arises from the properties of the energy level structure itself, such as the excited state absorption (ESA) mentioned in Section 3.3. In this framework, we recall that the largest differences among samples, as a function of individual nanoparticle size and morphology, were detected for the 532 nm laser wavelength, while at 355 nm, the properties appear less differentiated. This further confirms that the dominant mechanisms producing OL in these two spectral ranges are different. Moreover, it also confirms that, at 532 nm, the dominant mechanism involves the fundamental electronic properties of nanoparticles to a greater extent, which is in agreement with the ESA hypothesis in Section 3.3 on the basis of the concentration-dependent trend.

**Figure 11:**
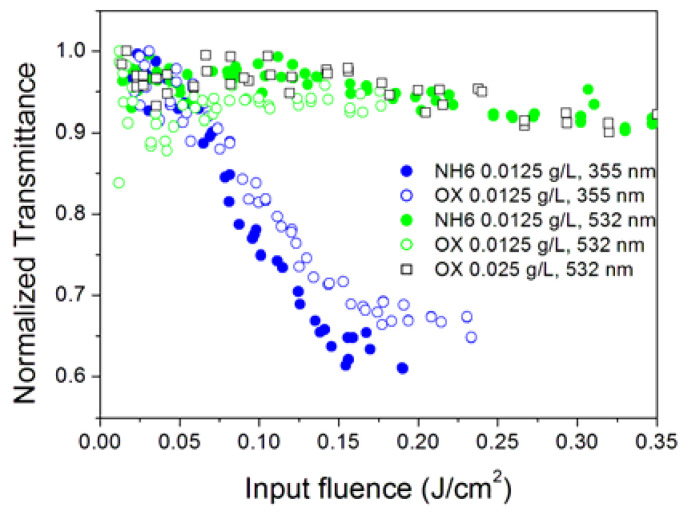
Optical limiting behavior of the NH6 and OX samples. It can be seen that the OX sample did not show optical limiting (OL) at 532 nm at the concentration of 0.0125 g/L (green hollow circles, no decrease in the transmittance with respect to the linear value).

Finally, it was also possible to compare the effect of functionalization of SWCNHs by oxidation. The oxidation process makes the nanofluids more stable over time, but it changes physical and chemical properties of nanoparticles and, consequently, it could change the interaction among them and with the base fluid. Figure 11 compares oxidized and pristine dahlia morphologies. When the comparison was carried out at the same 0.0125 g/L concentration (where, however, linear extinction coefficients of the OX sample are lower than those of NH6), at 532 nm, OX did not show optical limiting at all. If, however, we compare the NH6 sample at 0.0125 g/L with the OX sample at 0.025 g/L (thus, in this case, allowing a slightly higher extinction coefficient of OX than NH6), we are able to detect OL at 532 nm by the oxidized sample as well. It should be noted that OX (oxidized) and NH6 (pristine) samples are characterized by identical preparation methods and age. The different behavior at varying concentrations shows that some differences exist in the OL properties of the samples. The fact that these differences more clearly appear at 532 nm confirms their electronic origin. They can be due to a reduction in the oxidized sample of the interband π plasmon peak (π–π* transition), which is typical of SWCNH and other graphene-based nanostructures. This reduction of the plasmonic peak is probably ascribed to the presence of functional groups that are induced on the surface of the material and/or to partially damaged material due to oxidation process, as already observed in [33]. As for the 1064 nm wavelength, both samples are quickly damaged by the radiation, and the characterization was not possible. 

### 3.5. Effect of Sizes of Aggregates

**Figure 12:**
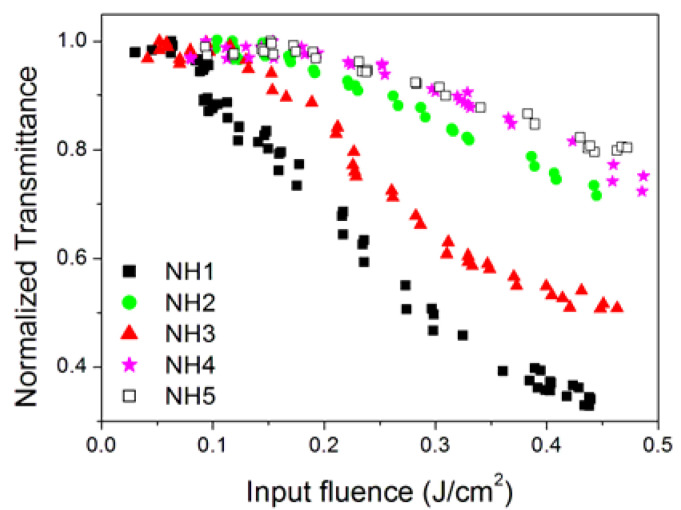
Comparison of OL performances at 532 nm and the concentration of 0.05 g/L as a function of the cluster size (see Table 2 for cluster dimensions).

A comparison of OL performances of samples with different sizes of aggregates, but with every other parameter (morphology and individual nanoparticle size) fixed, for the same 0.05 g/L concentration, and at a 532 nm wavelength is shown in Figure 12. 

The observed behavior is fairly correlated with the dimensions of aggregates: OL occurs at lower input energy density and with a higher slope as the dimensions of aggregates increase (see measured sizes in Table 2), similarly to what is reported in the literature for nanodiamond clusters [34]. The only exception to this trend is represented by sample NH5, which is characterized by partial damage of the nanohorn structure because of the prolonged homogenization process.

It is worth comparing here our results with the literature considering the NH4 sample, which is characterized by a similar hydrodynamic size with respect to the only available report (97 nm, see Table 2 vs. 95 nm as in [19]). For sample NH4 at the 532 nm wavelength, we observe that transmittance decreases to 90% of its linear value for input fluences of 0.1 J/cm^2^ for the lowest and 0.3 J/cm^2^ for the highest concentrations (Figure 5a), while in [19], the same transmittance value was obtained for input fluence of around 0.7 J/cm^2^. Considering the differences in the samples examined in the two works and the fact that in the cited reference several sample details are not given, the obtention of the same order of magnitude can be accepted as a fair agreement.

### 3.6. Modelling the Optical Limiting Curve

The appearance of OL in nanosuspensions can basically be explained by two mechanisms: nonlinear absorption and nonlinear refraction [35], depending on the nature of nanoparticles and on the properties of suspending fluids [6]. The effect that actually occurs can be identified by applying semi-empirical models. Nonlinear absorption can be due to different effects, such as reverse saturable absorption, two-photon absorption etc [6]. It can be modeled by introducing a nonlinear term in the Lambert–Beer law [7]:

(1) $$\frac{dF\left(z\right)}{dz}=-\alpha F-\frac{\alpha \sigma_{1}}{2\hbar \omega }F^{2}$$

where *F*(*z*) is the energy fluence at the *z* position in the sample, α is the linear extinction coefficient and, $\sigma_{1}$ is the excited state absorption (ESA) cross-section. If we solve this equation following the method described in [36,37], we can link the output energy from the nanofluid *E_out_* to incident energy *E_in_*, obtaining the following relationship:

(2) $$E_{out}=\frac{T_{s}^{2}e^{-\alpha L}E_{in}}{1+0.1\;E_{in}/E_{t}}$$

where $T_{s}^{2}$ is a coefficient describing the reflection losses on the walls of the cuvette, and *L* is the thickness of the sample. The term $e^{-\alpha L}$ describes the linear extinction, and $E_{t}$ is the threshold energy defined as the energy where the transmittance decreases to 90% of its original value [7]. In the following, we label this model as nonlinear Lambert–Beer (NL-LB).

On the other hand, a model where OL is only due to nonlinear refraction was developed in [38]. In the cited reference, nonlinear properties are ascribed to the scattering between laser photons and vapor bubbles yielded by the vaporization of the base fluid surrounding the nanoparticle and/or by ionization and vaporization of the nanoparticles:

(3) $$E_{out}=T_{s}^{2}E_{t}e^{-\alpha L}{\left(1+\frac{\alpha^{e}}{\alpha }\left(\frac{E_{in}}{E_{t}}-1\right)\right)}^{\frac{\alpha }{\alpha^{e}}}$$

where *α^e^* is the nonlinear extinction coefficient, and *E_t_* is the energy threshold for bubble formation, both obtained from a fitting procedure of experimental data. This model will be labelled as McEwan–Milsom–James (MMJ) in the following paragraph.

In order to verify whether theoretical models are compatible with our experimental data and, in the positive case, to estimate the values of the model parameters, we carried out a series of numerical tests. The procedure consisted of a standard least-squares approach, where the objective function is the sum *S* of the squared differences between the observed *E_out_* values and the corresponding computed values for each *E_in_*. The best fit model is that for which *S* is the minimum. In other terms, we look for the values of the model’s free parameters (*E_t_* and *T_s_* in the case of the NL-LB model, or *E_t_*, *T_s_* and *α^e^* in the case of the MMJ model) that cause *S* to be the minimum value (if they exist). The minimum value *S_min_* is a measure of the distance between the fittest theoretical model and the data set. Due to the fact that both *E_in_* and *E_out_* are affected by random errors, a χ^2^ standard approach cannot be straightforwardly followed for the evaluation of the statistical goodness of fit, expressed in terms of the probability *P* that, just by chance, we could have found a larger value of *S_min_*. In order to determine the correct statistics of *S_min_*, we followed a fully numerical Monte-Carlo approach consisting of randomly generating a number of simulated experimental sets based on the same theoretical model from our best fit, perturbing the values of *E_in_* and *E_out_* each time. For each simulated data set, we repeated the fit, counting the number of times we obtained a larger *S_min_*. If the probability P was less than 5%, we rejected the fit, declaring the model not compatible with experimental data. A similar Monte-Carlo approach was used to estimate the formal errors on the fitted model parameters. Both in the computation of the formal errors and of the goodness of fit, we assumed Gaussian random errors in *E_in_* and *E_out_* experimental values. 

**Figure 13:**
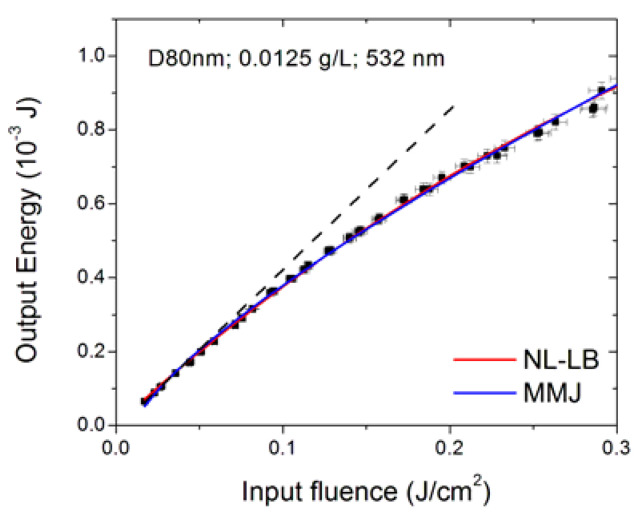
Fit of nonlinear Lambert–Beer (NL-LB) and McEwan–Milsom–James (MMJ) models on the optical limiting curve of theD80nm sample at 532 nm. The dashed black line is a visual reference for the linear regime.

**Figure 14:**
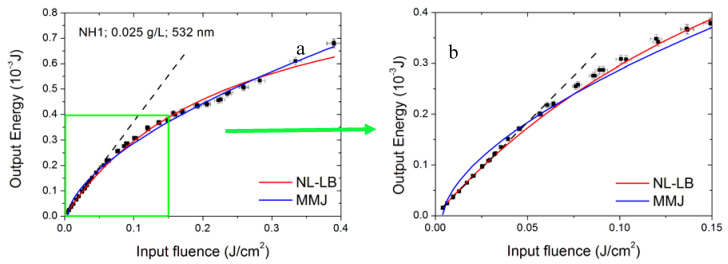
Fitting of the NH1 sample for the 532 nm laser wavelength and 0.025 g/L concentration. The plot (**b**) is an enlargement of the first experimental points on the (**a**) plot. The dashed black line shown in both plots is a visual reference for the linear regime.

As reported in Figure 13 for sample D80nm at 532 nm, when the optical limiting is not strong, both models are able to reconstruct the experimental data. For a fair comparison with both the data shown in the previous paragraphs and those reported in the literature, in the following, the fit results will be given in terms of fluence (energy density). Therefore, the obtained fluence threshold values in Figure 13 are (66 ± 4) ×10^−3^ J/cm^2^ for NL-LB and (44 ± 1) ×10^−3^ J/cm^2^ for MMJ models. On the other hand, if optical limiting is stronger, typically, the NL-LB model well reproduces the first experimental points deviating from linearity, while the MMJ model is more suited to reconstructing the final part of data points, as showed in Figure 14, depicting the NH1 sample for the 532 nm incident wavelength and 0.0025 g/L concentration. The plot on the right of Figure 14 is an enlargement of the first experimental points, showing that the NL-LB model better reproduces the experimental trend at lower input energy values, while the MMJ model is more appropriate for higher input ranges (Figure 14, left plot). For this specific case, the poor data reconstruction at low input energies prevents the MMJ model from being statistically acceptable (Table 4).

**Figure 15:**
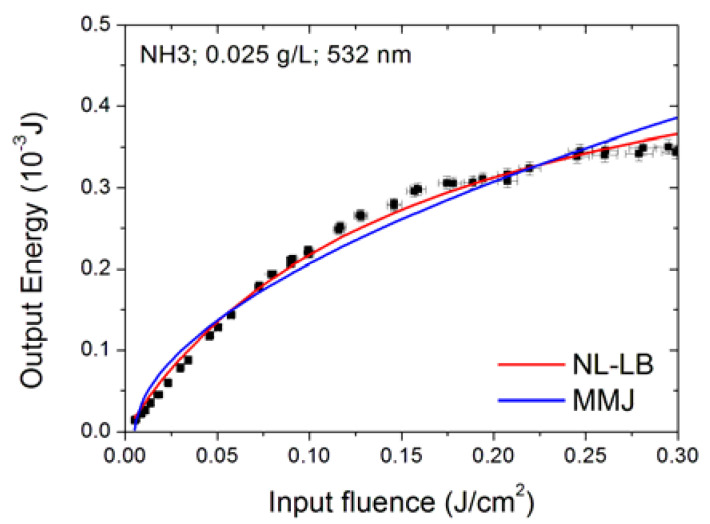
Strong OL in the NH3 sample for the 532 nm laser wavelength and 0.025 g/L concentration. Neither the NL-LB model (red curve) nor the MMJ model (blue curve) are able to reproduce the experimental data.

For all available data, both models were checked for fitting. The values of the obtained parameters for the different sample/concentration/wavelength combinations with a fixed statistical confidence level of 5% are listed in Table 4 for threshold fluence values *F_t_* = *E_t_/A*, with the laser spot area *A* and *E_t_* given by Equations (2) and (3) and in Table 5 for the *α^e^*/*α* ratio (Equation (3)). As previously mentioned, in some cases, both models are valid, while in other cases, only one of them is acceptable. Energy threshold fluence values are between 10^−3^ and 10^−1^ J/cm^2^. Typically, for a fixed sample and concentration, the fluence threshold increases with wavelength in agreement with the decrease in the linear extinction coefficient. Modelling OL data for the 1064 nm wavelength is a difficult task because of sample damage, and this is the reason why fitting results in the infrared are less represented in Table 4. In the majority of cases when fitting with both models is possible, MMJ thresholds are lower than those of NL-LB, suggesting the quicker onset of bubbles with respect to saturation phenomena. Further cases are found when none of the models give a reliable result. This happens, for instance, when a particularly strong OL is detected. These cases are characterized by a nearly constant output energy, irrespective of the input one, for input ranges above certain values, like the data shown in Figure 15. This strong OL behavior, also observed at the 355 nm wavelength for D80nm(0.025 g/L) and NH1(0.025 g/L) samples, is similar to that observed for glycol suspensions of graphite/nanodiamond mixtures [26] and is likely the result of a strong optical scattering due to a massive bubble production, stronger than that described for the MMJ model. In some other cases, the unsuccessful fitting of our models can be also due to the strong mixing between the two effects, which on the other hand, within each one of the considered semi-empirical models, are taken into account separately. These facts display the need to develop a comprehensive model that is able to include both nonlinear absorption and nonlinear refraction, which, however, is beyond the scope of the present work and will be the subject of further studies. 

**Figure 16:**
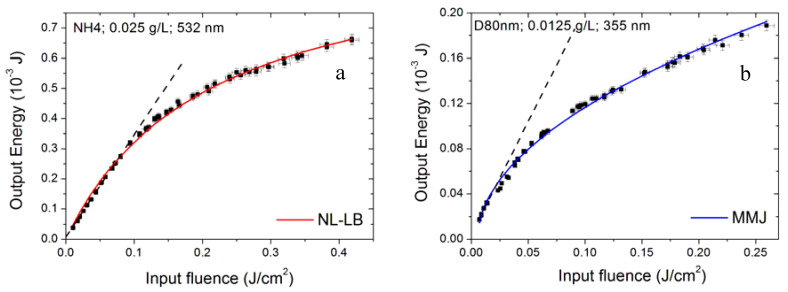
Examples of optical limiting data whose modelling can be univocally identified. The linearity regime is also depicted for reference (dashed black line) (**a**) NL-LB model; (**b**) MMJ model.

**Figure 17:**
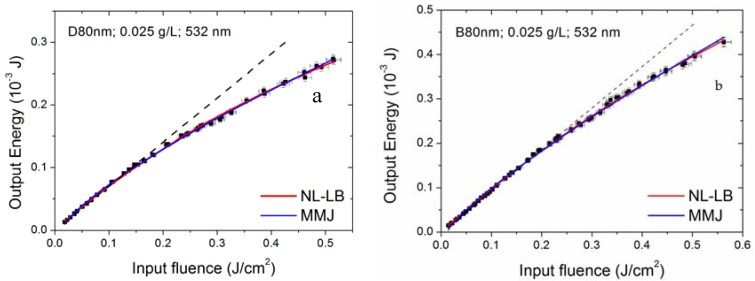
(**a**,**b**) Examples of optical limiting data allowing fitting by both models. The linearity regime (dashed black line) is also reported for enhanced visualization.

In Figure 16, we show some examples of data with univocal fitting, while Figure 17 shows some cases that can be modelled by both NL-LB and MMJ. It is worth noting that these models are able to numerically reconstruct a wide range of experimental data sets, including weaker OL regimes identified by the higher thresholds in Table 4. 

**Figure 18:**
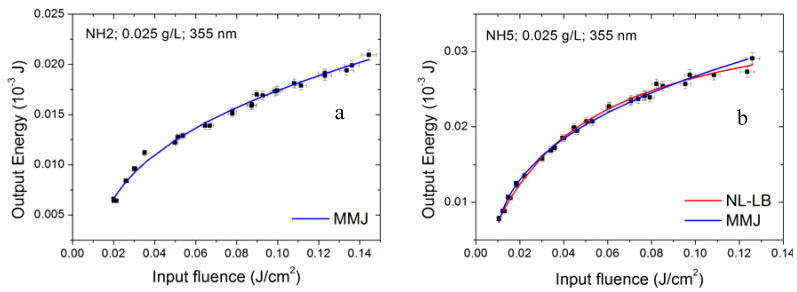
Optical limiting data and semi-empirical modelling for samples NH2 (**a**) and NH5 (**b**) under 355 nm radiation and at 0.025 g/L nanoparticle concentration.

The ratio *α^e^*/*α* between the nonlinear and linear extinction coefficient obtained from MMJ data fitting is summarized in Table 5. Its value in the majority of cases decreases with increasing laser wavelength and nanoparticle concentration. The highest obtained values are around 2.8 and 2.5 and were shown by the lowest concentration samples (NH5 and NH2) under 355 nm radiation. The relative fitting curves are shown in Figure 18.

### 3.7. Production of Vapor Bubbles

**Figure 19:**
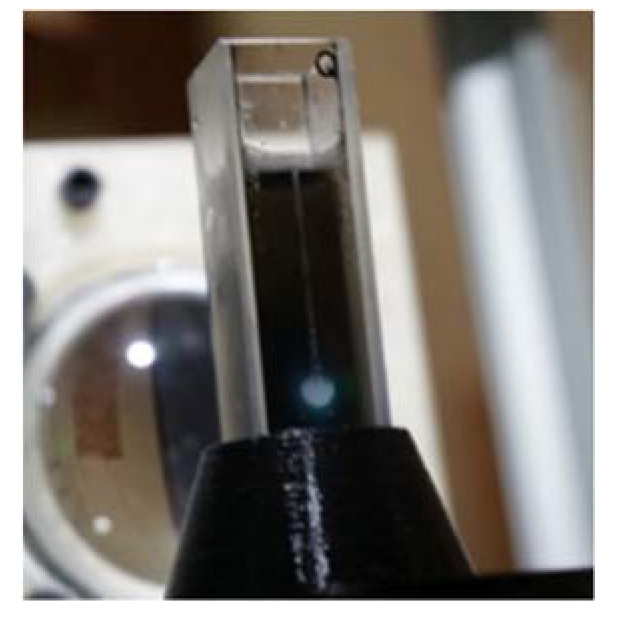
Production of vapor bubbles in NH1 samples under irradiation at 355 nm. The contrail of vapor rising up to the nanofluid surface is clearly visible above the white spot.

Bubble production has often been theorized in the literature [21,22] for carbon nanoparticles, but it has never been documented to date, except for a recent case investigated by our group on graphite/nanodiamond nanofluids dispersed in ethylene glycol [26]. We report in Figure 19 a photo of bubble production of our aqueous nanohorn-based nanofluids under 355 nm incident radiation. The laser beam immediately creates nano- and micro-bubbles that increase in volume due to bubble dynamics and coalescence. We can clearly see over the white spot a contrail of vapor rising up to the nanofluid surface. The video (Supplementary Material Video S1) recording of bubble formation is made available as additional material. To the best of our knowledge, it constitutes the first evidence of light irradiation-induced bubble generation in a water-based nanofluid. In fact, it is worth recalling that bubble formation critically depends on the system, being strongly related to thermophysical properties of the dispersing fluid, as well as optical and thermal properties of nanoparticles. We saw a massive bubble production for the 355 nm wavelength, while at 532 nm, the amount of produced bubbles was lower due both to the lower extinction coefficient and to the mechanism of saturation, which likely is the dominant one. No bubble production was observed for the 1064 nm wavelength for any SWCNH sample probably because of the quick sample damage.

## 4. Conclusions

The present work, using an original approach consisting of simple transmittance measurements joined to semi-empirical fitting models, proves nonlinear optical properties of SWCNH-based aqueous nanofluids at three different wavelengths (355, 532 and 1064 nm) and systematically investigates the dependence of optical nonlinearity from several sample parameters: surfactant concentration, nanoparticle concentration and morphology, dimensions of individual nanoparticles and dimensions of aggregates. The surfactant seems to have no impact on optical limiting, while dahlia morphologies appear to perform better than the bud one. Moreover, the starting size of individual nanoparticles, within the same dahlia-like morphology, appears to affect optical properties. Differences in optical limiting among pristine and oxidized dahlias are likely connected to the oxidation-induced reduction of the plasmonic peak. A cluster size effect is demonstrated for fixed morphology and individual nanoparticle size, showing that OL is more effective for larger aggregates. As for the effect of nanoparticle concentration, it is likely connected to the dominant mechanism producing optical limiting; at a 355 nm wavelength, OL is more effective (lower threshold and higher slope) for higher concentrations, while at 532 and 1064 nm wavelengths, it is more effective for lower concentrations. This dependence, together with the analysis of the nanoparticle size and morphology dependence of observed optical limiting, allows us to infer that the dominant mechanism at 355 nm is the generation of bubbles in the fluid, while at the other wavelengths, OL is mainly produced by saturation of absorption, even if, in the majority of cases, both effects appear to coexist. This is finally validated fitting experimental data by two models taking into account bubble formation and saturation, respectively. We also visually documented for the first time the generation of vapor bubbles in SWCNH samples under 355 nm radiation and, to a lower extent, under 532 nm radiation as well. The formation of bubbles is a key factor for possible future use of this type of nanoparticle in green energy, in particular as direct solar medium for vapor production. 

## Figures and Tables

**Table 1:** Investigated samples. All nanofluids, except oxidation (OX) ones, are prepared with not-functionalized (pristine) single-walled carbon nanohorns (SWCNHs). * Same protocol as NH2, prepared again for comparison with the OX sample at equal sample ages.

Sample Label	Morphology	Individual Nanoparticle (Dry) Size (nm)	Concentration g/L	Notes on Preparation
NH1	Dahlia	60	0.025 0.05	SDS 0.0025 g/L SDS 0.005 g/L Sonication only
NH2	Dahlia	60	0.025 0.05	SDS 0.0025 g/L SDS 0.005 g/L Sonication + 15′ homog.
NH3	Dahlia	60	0.025 0.05	SDS 0.0025 g/L SDS 0.005 g/L Sonication + 30′ homog.
NH4	Dahlia	60	0.025 0.05	SDS 0.0025 g/L SDS 0.005 g/L Sonication + 45′ homog.
NH5	Dahlia	60	0.025 0.05	SDS 0.0025 g/L SDS 0.005 g/L Sonication + 60′ homog.
NH6 *	Dahlia	60	0.0125	SDS 0.00625 g/L Sonication+15′ homog.
OX	Oxidized dahlia	60	0.0125 0.025	pH 4.8 Sonication + 15′ homog.
B80nm	Bud	80	0.0125 0.025 (0.05)	SDS 0.075 g/L SDS 0.15 g/L SDS 0.30 g/L
D80nm	Dahlia	80	0.0125 0.025	SDS 0.075 g/L 0.15 g/L

**Table 2:** Dimensions of aggregates and zeta potential of samples.

Sample Label	DLS Hydrodynamic Sizes (nm)	ζ Potential (mV)
NH1	202	−27.4
NH2	118	−29.0
NH3	151	−29.5
NH4	98	−32.7
NH5	123	−31.8
B80nm	197	−28.0
D80nm	241	−33.3
NH6	140	−46.5
OX	115	−45.1

**Table 3:** Obtained minimum transmittances at different wavelengths. The symbol “-” refers to the cases where the sample damage prevented quantitative measurements, and the symbol x to the cases of no optical limiting. For the 355 nm wavelength, due to the high linear absorption, which consistently reduced the amount of transmitted radiation below the detection limit of our apparatus, even in the linear regime, the characterization was possible only at the lowest concentration.

		Minimum Relative Transmittances T_min_		
Sample	Concentration g/L	@355 nm	@532 nm	@1064 nm
NH1	0.025 0.05	0.18	0.46 0.33	0.70 0.47
NH2	0.025 0.05	0.42	0.60 0.71	- -
NH3	0.025 0.05	0.24	0.43 0.49	0.83 0.80
NH4	0.025 0.05	0.30	0.44 0.63	0.82 -
NH5	0.025 0.05	0.35	0.56 0.65	- -
B80nm	0.0125 0.025	0.49 0.30	0.81 0.81	- -
D80nm	0.0125 0.025	0.31 0.24	0.75 0.73	- -
NH6	0.0125	0.64	0.90	-
OX	0.0125 0.025	0.70	x 0.90	- -

**Table 4:** Fluence threshold values obtained from fitting.

Sample	Concentration g/L	Model	Wavelength	Threshold Fluence (mJ/cm^2^)
**NH1**	**0.025**	NL-LB	532 nm	18.7 ± 0.8
		NL-LB	1064 nm	73 ± 10
**NH2**	**0.025**	MMJ	355 nm	21 ± 2
		NL-LB	532 nm	36.6 ± 1.2
	**0.05**	NL-LB	532 nm	64 ± 4
		MMJ	532 nm	133 ± 4
**NH3**	**0.025**	NL-LB	1064 nm	178 ± 21
**NH4**	**0.025**	NL-LB	532 nm	20.8 ± 0.8
		NL-LB	1064 nm	68 ± 5
	**0.05**	NL-LB	532 nm	70 ± 2
		MMJ	532 nm	128 ± 3
**NH5**	**0.025**	NL-LB	355 nm	4.0 ± 0.1
		MMJ	355 nm	12.6 ± 0.3
		NL-LB	532 nm	32.4 ± 0.8
	**0.05**	NL-LB	532 nm	93 ± 4
		MMJ	532 nm	125 ± 4
**B80nm**	**0.0125**	MMJ	355 nm	32 ± 1
		NL-LB	532 nm	145 ± 12
		MMJ	532 nm	46 ± 4
	**0.025**	NL-LB	532 nm	166 ± 17
		MMJ	532 nm	64.5 ± 0.8
**D80nm**	**0.0125**	MMJ	355 nm	11.1 ± 0.3
		NL-LB	532 nm	66 ± 4
		MMJ	532 nm	44 ± 1
	**0.025**	NL-LB	355 nm	7.0 ± 0.7
		NL-LB	532 nm	108 ± 8
		MMJ	532 nm	48 ± 1
**NH6**	**0.0125**	NL-LB	355 nm	20 ± 1
		MMJ	355 nm	11.9 ± 0.3
		NL-LB	532 nm	832 ± 416
**OX**	**0.0125**	NL-LB	355 nm	34 ± 2
		MMJ	355 nm	29 ± 3

**Table 5:** Ratio of nonlinear-to-linear extinction coefficients from MMJ model fitting.

Sample	Concentration g/L	*α^e^*/*α*	Wavelength
**NH2**	**0.025**	2.5 ± 0.2	355 nm
	**0.05**	2.0 ± 0.2	532 nm
**NH4**	**0.05**	1.9 ± 0.1	532 nm
**NH5**	**0.025**	2.8 ± 0.2	355 nm
	**0.05**	1.6 ± 0.1	532 nm
**B80nm**	**0.0125**	2.3 ± 0.1	355 nm
		1.2 ± 0.1	532 nm
	**0.025**	1.2 ± 0.1	532 nm
**D80nm**	**0.0125**	2.0 ± 0.1	355 nm
		1.4 ± 0.1	532 nm
	**0.025**	1.3 ± 0.1	532 nm
**NH6**	**0.0125**	1.5 ± 0.1	355 nm
**OX**	**0.0125**	1.5 ± 0.1	355 nm

**Figure A1:**
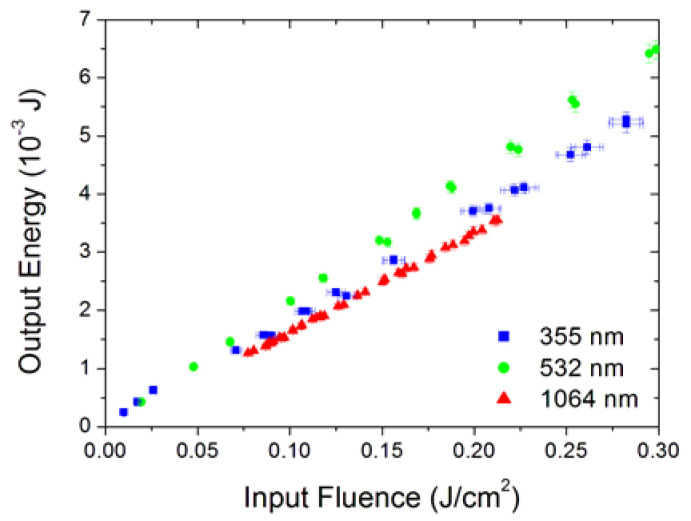
Linearity of the base fluid (water + surfactant).

## Supplementary Materials

The following are available online at https://www.mdpi.com/2079-4991/10/11/2160/s1, Video S1: Video recording of bubble generation in the NH1 sample at a concentration of 0.025 g/L and a 355 nm laser wavelength.

## Abbreviations

**Nomenclature**	
***Abbreviations***	
OL	Optical limiting
CNTs	Carbon Nanotubes
SWCNHs	Single Walled Carbon Nanohorns
SDS	Sodium Dodecyl Sulphate
NH1, NH2, NH3, NH4, NH5, NH6, OX, B80nm, D80nm	Sample labels (explanations in Table 1)
UV–VIS	Ultraviolet–Visible
UV	Ultraviolet
DLS	Dynamic Light Scattering
ESA	Excited State Absorption
SEM	Scanning Electron Microscope
TEM	Transmission Electron Microscope
XRD	X-ray Diffraction
NL-LB	Nonlinear Lambert–Beer
MMJ	McEwan–Milsom–James
***Symbols***	
*E*	Energy
*F*	Energy fluence
*T*	Relative transmittance
ħ	Reduced Planck constant
*ω*	Angular speed of the electromagnetic wave
*z*	Position in the sample
*α*	Linear extinction coefficient
*σ*_1_	Excited State Absorption cross section
*T_s_* ^2^	Fitting coefficient (see text for explanation)
*L*	Sample thickness
*α^e^*	Nonlinear extinction coefficient
*S*	*S* = Σ*_i_* (observed *E_out, i_* − computed *E_out, i_*)^2^, see text for explanation
*A*	Laser spot area
***Subscripts***	
in	input
out	output
m	minimum
t	threshold

## Appendix A. Linearity of the Base Fluid

To exclude the notion that the observed OL in SWCNH-based nanofluids could be due to SDS surfactant rather than to nanoparticles, we prepared a solution of water and SDS without nanoparticles to also be subjected to optical limiting experiments.

To detect even very small OL effects produced by SDS, the SDS concentration in the sample without nanoparticles was 0.5 g/L, much higher than in all the samples laden with SWCNHs (Table 1), and the investigation was carried out exploring, for the different wavelengths, the energy range where OL was previously obtained in the cases of nanofluids, before their damage. As shown in Figure A1, at all wavelengths, the base fluid had a completely linear behavior. This allowed us to conclude that the OL effects we obtained for SWCNH-nanofluids arose from nanoparticles.
